# Fabrication of Polycaprolactone/Polyurethane Loading Conjugated Linoleic Acid and Its Antiplatelet Adhesion

**DOI:** 10.1155/2017/5690625

**Published:** 2017-05-16

**Authors:** Ho Hieu Minh, Nguyen Thi Hiep, Nguyen Dai Hai, Vo Van Toi

**Affiliations:** ^1^Tissue Engineering and Regenerative Medicine Laboratory, Department of Biomedical Engineering, International University of Vietnam National Universities, Ho Chi Minh City 700000, Vietnam; ^2^Institute of Applied Materials Science, Vietnam Academy of Science and Technology, 01 Mac Dinh Chi, District 1, Ho Chi Minh City, Vietnam; ^3^Graduate University of Science and Technology, Vietnam Academy of Science and Technology, Hanoi, Vietnam

## Abstract

Polycaprolactone/polyurethane (PCL/PU) fibrous scaffold was loaded with conjugated linoleic acid (CLA) by electrospinning method to improve the hemocompatibility of the polymeric surface. Fourier Transform Infrared Spectroscopy (FT-IR) analysis and Scanning Electron Microscopy (SEM) observation were employed to characterize the chemical structure and the changing morphology of electrospun PCL/PU and PCL/PU loaded with CLA (PCL/PU-CLA) scaffolds. Platelet adhesion and whole blood clot formation tests were used to evaluate the effect of CLA on antithrombotic property of PCL/PU-CLA scaffold. Endothelial cells (EC) were also seeded on the scaffold to examine the difference in the morphology of EC layer and platelet attachment with and without the presence of CLA. SEM results showed that CLA supported the spreading and proliferation of EC and PCL/PU-CLA surface induced lower platelet adhesion as well as attachment of other blood cells compared to the PCL/PU one. These results suggest that electrospinning method can successfully combine the antiplatelet effects of CLA to improve hemocompatibility of PCL/PU scaffolds for applications in artificial blood vessels.

## 1. Introduction

Various approaches have been investigated to improve blood compatibility of polymeric surfaces for artificial blood vessels (ABVs) application including chemical and biological modification [1, 2]. However, polymeric materials for small ABVs are still undergoing investigation and most of them induce thrombosis [3], infection, and calcination [4]. Among these, the main cause of failure in ABVs is thrombosis [5]. When implanted inside human body, polymeric materials exposed to physiological fluids can initiate a complex cascade of surface induced thrombotic events [6] as follows: adhesion and activation of platelets result in liberation of different agents such as adenosine diphosphate (ADP), arachidonic acid (AA), thromboxane A_2_ (TXA_2_), and thrombin, which help circulating fibrinogen bind to platelet scaffold and connect platelets together [7]. The activities of these aggregating agents act as signal for further platelets' activation and aggregation to form a loose plug at wounded area. Then thrombin converts fibrinogen into fibrin to form a mesh-like stable plug, which is called a thrombus.

As explained above, fibrinogen is a key factor in thrombus formation. Fibrous coagulant in the blood significantly increases the risk of cardiovascular diseases, one of the leading causes of death and disabilities including hypertension, ischemia, myocardial infarction, stroke, and limb loss. Platelets also have a very important role in hemostasis and thrombogenesis. Platelet is a catalyzing coagulation reaction agent, which leads to the formation of fibrin, but does not react negatively with other blood cells [8]. Therefore, one of the requirements of polymeric materials for ABVs application is antithrombus property, including antiplatelet adhesion, and reduces blood protein (fibrinogen) attraction.

To enhance antithrombotic property, ABVs polymeric surfaces can be modified to become very hydrophobic (e.g., PDMS graft) [9] or hydrophilic (e.g., PEG graft) [10, 11] or using anticoagulation agents such as heparin [12] and albumin [13]. There is another antiplatelet agent, CLA, which can improve the hemocompatibility of biomaterials. In 2006, CLA grafted to polyacrylonitrile (PAN-CLA) to improve their hemocompatibility was investigated by Kung et al. The result showed that PNA-CLA offered a new composite for hemodialysis [14, 15]. CLA is both a cis and trans unsaturated fatty acid and has been known as famous drug for antithrombus [16]. CLA was found as inhibitor of platelet aggregation induced by different aggregating agents such as collagen, ADP, AA, and thrombin [17]. CLA was also found to decrease activity of cyclooxygenase-1 (COX_1_), an enzyme which converts AA into TXA_2_, resulting in opposition to platelet's aggregation [18].

In our previous study, a hybrid electrospun PU/PCL scaffold satisfying the requirements of blood vessel prosthesis with suitable mechanical properties, sufficient pore size (ranging from 5 to 150 *µ*m) for nutrient diffusion, and high biocompatibility was successfully fabricated [19]. The result showed that the PCL/PU scaffold had good biocompatibility and mechanical properties. Blend PCL/PU composite has high potential for ABV scaffold because PCL provides favorable EC attachment and proliferation [20] combined with high tensile strength, pressure strength, and blood compatibility (blood-contacting devices) of PU [21]. In continuation for this research, the electrospun PCL/PU scaffold was loaded with CLA due to its inhibitory effects on platelet function which is expected to offer a new hemocompatibility component for ABV applications. Electrospinning is a technique that can produce polymeric fibers from polymer solution by using electric force and heat to drive the spinning process [22]. Electrospinning is one of the simplest among all methods for preparation of fibrous mat used widely for a lot of biomedical applications [23]. Electrospun fibers not only mimic extra cellular matrix but also play another role as carrier of anticoagulant agent like in this investigation and were found to support mechanical and burst strength for ABVs in our previous paper. Several methods can be used to introduce CLA to PCL/PU scaffold such as blending, coating, and grafting [24]. However, blending was chosen due to its simplicity and not requiring complicated apparatus.

The ultimate goal of this investigation was to employ electrospinning method to fabricate electrospun PCL/PU loading CLA scaffolds for ABV applications. The direct addition of CLA to the PCL/PU blend is attempted to enhance blood compatibility of polymeric materials implanted inside human body. Hemocompatibility of electrospun PCL/PU and PCL/PU-CLA scaffolds was evaluated with blood clotting and platelet adhesion tests using fresh human blood.

## 2. Experimental Procedure

### 2.1. Materials

As starting materials, polyurethane (PU, Sigma), polycaprolactone (PCL, Mn 80,000, Sigma), conjugated linoleic acid (CLA, Sigma), tetrahydrofuran (THF, minimum 99%, Sigma), dimethylformamide (DMF, 99%, Sigma), ethanol (EtOH, 99%, Merck), and phosphate buffer saline (PBS, GIBCO) were used.

### 2.2. Preparation of PU/PCL and PU/PCL-CLA Electrospun Fibrous Scaffolds

PCL/PU and PCL/PU-CLA scaffolds were fabricated as mentioned in our previous study [19]. In brief, electrospinning of PCL/PU and PCL/PU-CLA scaffolds was fabricated by 12 wt% of blend PCL : PU (1 : 1) in DMF : THF (1 : 1) solution. Then, 0.2 ml CLA was added to 10 ml polymer solution. The solution was stirred for 12 hours in room temperature until azeotrope solution. The solutions were electrospun directly with 27 kV power supply (NNC-30 kV-2 mA portable type, Korea) using vertical electrospinning setup. A grounded steel cylinder, 15 cm away from the tip of the syringe needle (ID = 0.25 mm), was used for collection of the nanofiber mats. Flow rates of the PCL/PU solutions (0.5 ml/hour) were controlled by syringe pump (luer-lock type, Korea).

### 2.3. Structure Characterization

#### 2.3.1. Morphology Analysis

Morphology of electrospun PCL/PU and PCL/PU-CLA fibrous mats was observed by SEM (JEOL JSM-IT100, Japan) with gold sputter coating (JEOL Smart Coater, Japan).

#### 2.3.2. Fourier Transform Infrared Spectroscopy (FT-IR)

Electrospun PCL/PU and PCL/PU-CLA mats were characterized by attenuated reflectance Fourier transform spectroscopy (Spectrum GX, PerkinElmer, USA). The infrared spectra of the samples were measured over a wavelength range of 4,000–400 cm^−1^. All spectra were taken in the spectral range by the accumulation of 64 scans with a resolution of 4 cm^−1^.

### 2.4. Hemocompatibility Testing

For this test, specimens of sample were fixed (circle shape, *R* = 1 cm).

#### 2.4.1. Platelet Adhesion Test

Platelet adhesion test was done on four types of scaffolds: EC seeded (PCL/PU/EC and PCL/PU-CLA/EC) and non-EC seeded (PCL/PU and PCL/PU-CLA) before addition of platelets. The purpose of seeding EC on these scaffolds is based on the fact that when ABVs are implanted in human body, migration, adhesion, and proliferation will occur creating a single layer of EC on ABVs surface. Therefore the difference in antithrombotic property of scaffolds with and without presence of EC must be investigated. For preparation of cell-seeded scaffolds, healthy EC were prepared carefully by changing media regularly every 2 days and keeping them below 90% cell confluence. Then EC were washed by PBS and detached by trypsin-EDTA. Addition of fresh media was done to make a suspension of cells and the pellet was obtained after centrifuging and was resuspended. The final suspension was used for seeding cells. 10^5^ of EC were seeded on 1 cm^2^ electrospun scaffolds which had been precultured for 14 days before using in platelet adhesion testing. The cultured media was changed every 3 days to obtain healthy sheet of EC on electrospun scaffolds.

For preparation of platelets, a volume of 180 ml fresh human blood (approved and supplied by Hospital Ethics Committee of Blood Transfusion Hematology Hospital, Ho Chi Minh City, Vietnam) containing 20 ml of 3.8 v/v% sodium citrate in PBS solution as anticoagulant (sodium citrate: blood ratio, 1 : 9) was centrifuged at 4°C, 700 g, for 20 min [25]. Electrospun PCL/PU and PCL/PU-CLA scaffolds (with and without EC seeding) were equilibrated with PBS overnight before immersing in platelets at 37°C with shaking (100 rpm) in an incubator. After 8 h of incubation, the samples were taken out, rinsed five times with PBS, and fixed by immersing in 2% v/v glutaraldehyde in PBS solution at room temperature for 2 h. After fixation, the samples were dehydrated with a series of ethanol solutions (50, 60, 70, 80, 90, and 100 v/v%) for 15 min per each step. Then they were dried in atmosphere overnight. The dried samples were coated with evaporated gold, and the adherent platelets were observed with SEM (JEOL, Japan).

#### 2.4.2. Whole Blood Clotting Formation

After washing thoroughly with PBS, PCL/PU and PCL/PU-CLA scaffolds were immersed twice in citrated human whole blood. Then the scaffolds were washed again with PBS before being incubated in recalcified whole blood (citrated human whole blood which contained 0.010 M CaCl_2_) for 20 min at room temperature. Prior to this testing, these surfaces were washed extensively with PBS until a prothrombin time for a dilution of the PBS wash into plasma was observed to be normal compared to controls [26] and then examined for the presence of thrombus by camera (Nikon P90). For SEM observation, the adsorbed whole blood on the electrospun scaffolds was rinsed five times with PBS and fixed by immersing the sample in 2 v/v% glutaraldehyde in PBS solution at room temperature for 2 h. After that fixation, the samples were dehydrated as foresaid. Then the dried samples were observed with SEM (JEOL, Japan).

## 3. Results and Discussion

**Figure 1:**
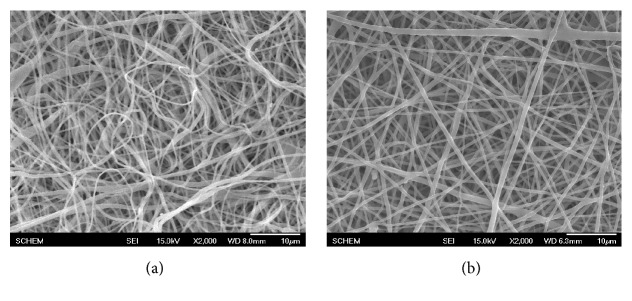
SEM morphology of electrospun PCL/PU (a) and PCL/PU-CLA (b) scaffolds.

Although CLA has been known to possess antithrombotic properties both in vitro and in vivo for many years, very little research have developed CLA as an additive to increase hemocompatibility of ABVs. Furthermore, the number of methods used to immobilize CLA onto ABVs' surface is still very limited, including esterification [27] and grafting [14]. In our previous study, electrospinning method was chosen to fabricate PCL/PU scaffold and in our current study CLA loading onto the membrane is carried out due to its advantage in controlling chemical ratios and examining the variation for different types of scaffolds.  Figure 1 shows the difference of SEM morphology between electrospun PCL/PU (Figure 1(a)) and PCL/PU-CLA (Figure 1(b)) fiber mats. The fiber's diameters in both types of scaffolds are approximately 1 *µ*m; however the number of fibers with such diameter size is greater for PCL/PU-CLA scaffold than the PCL/PU one. In addition, comparing the two morphologies, PCL/PU-CLA fibers were distributed with linear pattern while PCL/PU fibers convolute on top with diameter size varying in a wide range. Addition of CLA to PU/PCL solution electrolytes increases the electrical conductivity of polymer solution. That explains why average diameter size for PCL/PU-CLA fibers is smaller (1 *µ*m) while the number of fibers at such diameter size for PCL/PU scaffold is less. This agrees with what was found in Angammana and Jayaram report: average fiber diameter decreases with the increase in electrical conductivity of the solution [28].

**Figure 2:**
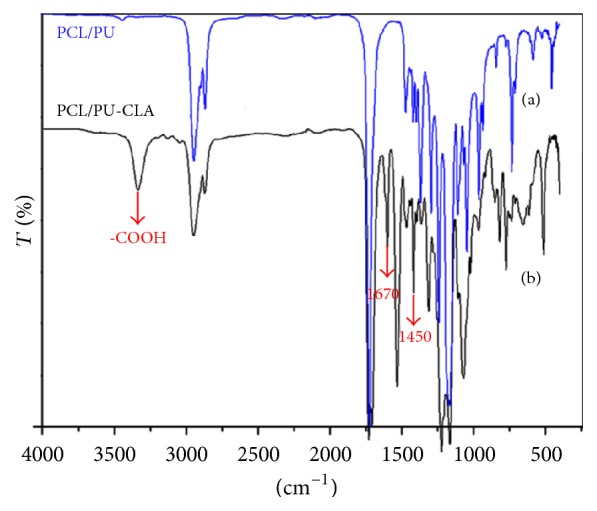
FT-IR spectrum of electrospun PCL/PU (a) and PCL/PU-CLA (b) scaffolds.

The existence of CLA loaded on PCL/PU scaffold was confirmed by employing FT-IR analysis. Typical FT-IR spectra of electrospun PCL/PU and PCL/PU-CLA mats are shown in Figure 2. From electrospun PCL/PU mat (Figure 2(a)), peaks appearing for PU hard segments are observed at 3320 cm^−1^ (urethane N-H, stretch), 1735 cm^−1^ (urethane C=O, free from hydrogen bonding), 1710 cm^−1^ (urethane C=O, hydrogen bonded), and 1535 cm^−1^ (C-N-H, bending) [29]. The characteristic absorbance of ester in PCL is shown at 1734 cm^−1^ [30]. However, peaks 1670 cm^−1^ and 1450 cm^−1^ are detected due to the C=C and C=O groups of CLA [14] in the FT-IR spectrum of the PCL/PU-CLA mat (Figure 2(b)), apart from peaks of PU and PCL as described above. In addition, an obvious peak is also found at 3300 cm^−1^ representing the O-H stretch in carboxyl group of CLA. Thus it proves that PCL/PU scaffold loaded with CLA was fabricated successfully by using electrospinning method.

**Figure 3:**
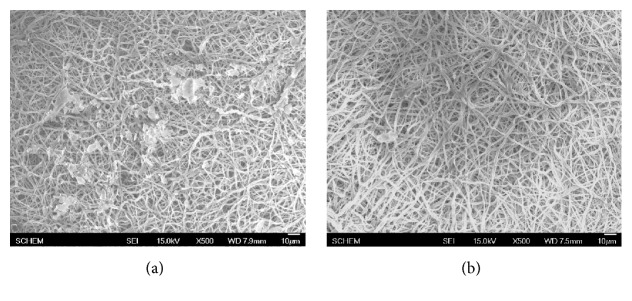
SEM morphology of platelets adhered on electrospun PCL/PU (a) and PCL/PU-CLA (b) scaffolds.

**Figure 4:**
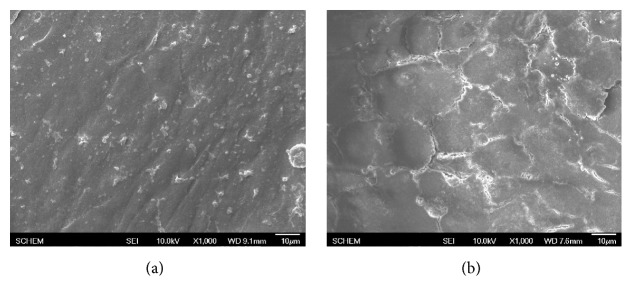
SEM morphology of platelets adhered on electrospun PCL/PU (a) and PCL/PU-CLA (b) scaffolds seeded with endothelial cells for 14 days.

Platelet adhesion on ABVs' surface is an essential test in evaluation of hemocompatibility of biomaterials as platelets play the main role in the formation of blood clot. In this study, we performed various simple tests which used both platelet rich plasma to examine the effect of PCL/PU-CLA scaffold on platelet function and human fresh whole blood to evaluate the adhesion of other blood cells. Interaction of platelets with electrospun PCL/PU and PCL/PU-CLA surfaces was examined using platelet rich plasma (PRP) prepared from human whole blood (Figures 3 and 4). In Figure 3, SEM morphology images show that platelets deposited and aggregated onto the fibrous PCL/PU scaffold (Figure 3(a)) while electrospun PCL/PU-CLA scaffold exhibits little platelet adhesion (Figure 3(b)). Based on the SEM images, electrospun PCL/PU-CLA displayed high antiplatelet activity with nonplatelet adhesion. In addition, to mimic the conditions of human body, endothelial cells were seeded on the scaffold and the difference in the effect of CLA with and without EC was also examined. To examine antiplatelet ability after the formation and proliferation of EC, PCL/PU and PCL/PU-CLA scaffolds were seeded with EC and the results of platelet adhesive behavior of EC seeded scaffolds are shown in Figure 4. To ensure that EC covered the scaffold surface, electrospun PCL/PU and PCL/PU-CLA were incubated for 14 days before testing. The results show that electrospun PCL/PU scaffold was good for EC spreading and elongation, the sheet of EC covered with dense surface. However, electrospun PCL/PU/EC also attracted platelet adhesion as shown in Figure 4(a) with distribution of platelets on EC layer. In contrast, although electrospun PCL/PU-CLA scaffold did not create an excellent sheet of EC as compared to the PCL/PU, platelet adhesion was not found on electrospun PCL/PU-CLA/EC scaffold (Figure 4(b)).

**Figure 5:**
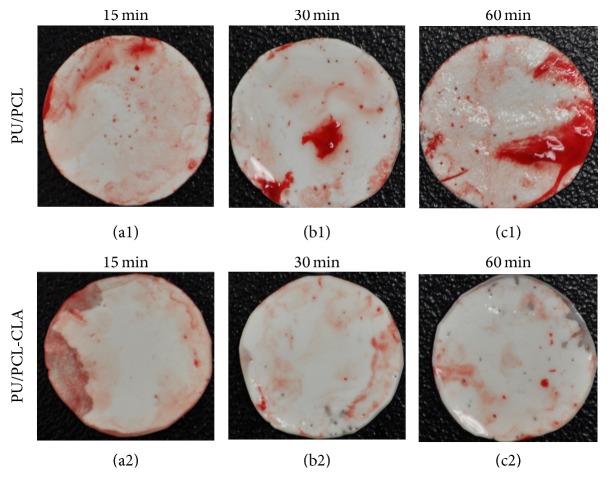
Photographs of whole blood clotting on electrospun PCL/PU (a1, b1, and c1) and PCL/PU-CLA (a2, b2, and c2) scaffolds for 15, 30, and 60 minutes.

**Figure 6:**
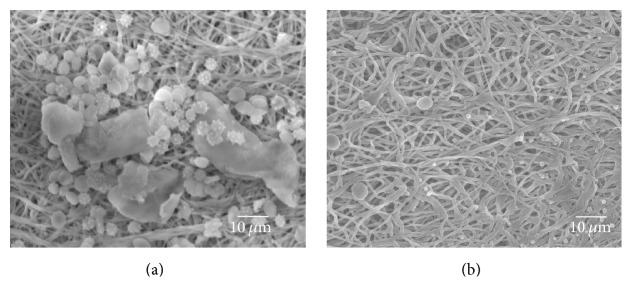
SEM morphology of whole fresh blood adhered on electrospun PCL/PU (a) and PCL/PU-CLA (b) scaffolds.

The initial step of platelet adhesion in thrombogenesis is very important and determines mural thrombosis that occurs assisted by other blood cells in later steps [8]. To make sure CLA has the ability of antithrombus, electrospun PCL/PU and PCL/PU-CLA scaffolds after incubation in whole fresh blood were washed thoroughly in PBS for detection of thrombus. The result shows that thrombus formation is reduced at test times of 15, 30, and 60 mins on PCL/PU-CLA scaffold as compared to the PCL/PU one (Figure 5). This proves that CLA were loaded successfully and supported electrospun PCL/PU scaffold improving its antithrombogenic activity. Under the same conditions, red blood cells may comprise a large proportion of total thrombus mass and contribute to chemical factors that influence platelet reactivity [8].  Figure 6(a) shows that electrospun PCL/PU scaffold induced platelet adhesion resulting in higher level of deposition and aggregation of red blood cells and white blood cells on the scaffold. Meanwhile, electrospun PCL/PU-CLA scaffold did not attract platelets and only a few blood cells were observed (Figure 6(b)).

The results show that electrospun PCL/PU-CLA scaffold significantly reduces the adhesion of platelets and almost no red or white blood cells as well as no thrombus are found on the scaffold. In addition, although CLA was shown to have less support for the proliferation of EC, its antiplatelet property was retained with the presence of EC on the scaffold. These findings confirm again the antiplatelet and antithrombotic properties of CLA, suggesting that CLA can be used as a new additive agent to increase hemocompatibility of ABVs and therefore decrease the amount of anticoagulant injected to human body after the implantation. From this study, electrospinning is also proved to be effective in PCL/PU scaffold fabrication and loading CLA as anticoagulant agent, which offers a new direction for different research on blood-contacting polymeric biomaterials.

## 4. Conclusion

In this study, PCL/PU scaffold loaded with CLA was electrospun as substrate for ABV applications. FT-IR results demonstrated that CLA was successfully immobilized in PCL/PU blend. Electrospun PCL/PU-CLA fibers were also found to be smoother and bind to each other as compared to those without CLA. Hemocompatibility tests showed that PCL/PU-CLA scaffold significantly decreased platelet adhesion and thrombus formation with no attachment of red and white blood cells. SEM morphology of endothelial cell layer on both PCL/PU and PCL/PU-CLA scaffolds demonstrated that CLA had less support of EC spreading and elongation but the antiplatelet property of CLA was retained with the presence of EC. These results show that CLA enhances hemocompatibility of PCL/PU scaffold in terms of platelet adhesion and thrombus formation. Further research which focuses on investigating CLA concentration to optimize the anticoagulant effect of CLA in PCL/PU scaffold needs to be conducted before being used in human body.
